# Variance-Triggered Two-Step GPS Acquisition

**DOI:** 10.3390/s19143177

**Published:** 2019-07-19

**Authors:** Fabrício Costa, Glauberto Leilson Albuquerque, Luiz Felipe Silveira, Carlos Valderrama, Samuel Xavier-de-Souza

**Affiliations:** 1 Departamento de Computação e Automação (DCA), Universidade Federal do Rio Grande do Norte, Natal 59078-970, Brazil; 2 Service d’électronique et de Microélectronique (SEMi), Universitè de Mons, 7000 Mons, Belgium

**Keywords:** GPS acquisition, variance-based detection threshold, two-step acquisition

## Abstract

The acquisition is the most time-consuming step performed by a Global Navigation Satellite System (GNSS) receiver. The objective is to detect which satellites are transmitting and what are the phase and Doppler frequency shift of the signal. It is the step with the highest computational complexity, especially for signals subjected to large Doppler shifts. Improving acquisition performance has a large impact on the overall performance of the GNSS reception. In this paper, we present a two-step Global Positioning System (GPS) acquisition algorithm whose first step performs an incremental correlation to find a coarse pair of phase and frequency and the second step, triggered by the variance of the largest correlation values, refines the first step. The proposed strategy, based on the conventional time-domain serial algorithm, reduces the average execution time of the acquisition process to about 1/5 of the conventional acquisition while keeping the same modest logic hardware requirements and slightly better success and false-positive rates. Additionally, the new method reduces memory usage by a factor that is proportional to the signal’s sampling frequency. All these advantages over conventional acquisition contribute together to significantly improve the overall performance and cost of GPS receivers.

## 1. Introduction

The first step performed by a Global Navigation Satellite System (GNSS) receiver is the acquisition. It consists of roughly finding the phase and frequency of an incoming GNSS signal and identifying the transmitting satellite. The phase of the signal is never known prior to acquisition and, as a result of the Doppler effect, the incoming frequency changes over time. The acquisition procedure should, therefore, explore a wide spectral range to detect phase and frequency of the signal and determine which satellites are transmitting by testing all plausible hypotheses in a bi-dimensional search space.

Frequency domain acquisition strategies have gained much attention [1,2]. This type of acquisition uses a Fast Fourier Transform (FFT) block to test all phases at once while iterating over the Doppler shift space sequentially. A recent two-step method, called Multi-Peak Double-Dwell, performs the acquisition using the FFT with a coarse step and a fine step [3]. Although FFT-based methods are usually faster than conventional time domain strategies, they require supplementary hardware, which increases the project requirements and cost [4]. Thus, conventional Serial Acquisition (SA) approaches that consider the reduction of the acquisition time without compromising the hardware simplicity are very desirable. Many receiver designs explore acquisition in the time domain associated with a strategy to improve performance. In [5], a folding technique exploits the sparsity of the GNSS signal to reduce computational complexity. In this technique, the signal is folded and the correlation is performed on the folded signal. Although computationally more efficient than SA, this method is more sensitive to the carrier-to-noise ratio (C/N0) degradation caused by an increase in the number of folds [5]. The eXtended replica Folding Acquisition Search Technique (XFAST) [6] is another folding strategy that directly reduces the code phases to look for by folding the local signal. Nevertheless, after folding, the correlation properties of the signal are degraded; hence, the detection performance for weak signals is worse than that of non-folding methods [5]. The Deterministic Compressed Sensing (DCS) is another acquisition technique based on a folding strategy that can achieve a better response in lower C/N0 [7] in comparison to conventional folding techniques. This technique also exploits the sparsity of a signal to reduce the number of measurements required for successful representation of the signal. The DCS is related to the problem of finding sparse solutions to vastly under-determined systems of linear equations. There are also significant connections with the problem of recovering signals from highly incomplete measurements [8,9,10]. The main contribution of DCS acquisition is the use of an appropriate transformation matrix to most sparsely express the target signal and to develop a measurement matrix along with an associated efficient reconstruction algorithm. Even though this technique achieves higher noise robustness than conventional folding techniques, it still suffers from poor accuracy at low C/N0.

In addition to the folding approaches that target the phase search, some techniques exploit the size of the frequency bin to improve acquisition time. In [11], a proof of the influence of the frequency bin size on the overall performance of acquisition is presented. It is shown that small Doppler bins increase the probability of a global false-positive, but increase the detection probability within the correct Doppler bin.

The above mentioned time-domain techniques search for the phase and frequency pair one after each other, using all signal samples available, folded or not. This means that when the correct pair is the last one to be tested, independently of the signal quality, all other pairs had already failed, resulting in the worst case complexity. However, even for the average case complexity, when the correct satellite pair is midway through the list of pairs to be tested, it might not be necessary to use all available samples if the quality of the signal is sufficiently high. We argue that it is possible to detect higher quality signals faster by testing all pairs incrementally for a group of samples.

This paper extends two partial results [12,13] to propose a folded Variance-Triggered Two-Step (VTTS) acquisition method in the time domain that presents a reduced acquisition time while keeping the same hardware requirements and slightly better success and false-positive rates when compared to the conventional serial acquisition. Using the same arithmetic operations, the new method implements an adaptive folding that allows the complexity to vary according to the C/N0 quality of the signal at the receiver. The higher the quality of the signal, the shortest the time required for acquisition. The variable complexity arises from the transition between the two steps of the approach, that alternate between a coarse search and a fine search until the specified satellite is acquired or defined as not present. To enable this transition to happen more efficiently, we have proposed a detection criterion to trigger the fine search step from the coarse search step. The proposed strategy allows a reduction of about 80% of processing time in comparison to the conventional strategy. In addition, the use of the proposed variance-based detection threshold provided faster acquisition and lower false-positive rate when compared to the use of ratio detection [14]. The proposed method also uses less memory than the conventional acquisition at a reduction factor that is proportional to the number of samples per chip or, equivalently, proportional to the sampling frequency.

This paper is organized as follows. In the next section, we provide background about the conventional acquisition. Then, we present the proposed two-step acquisition technique. The experiments we carried out to analyze and validate the proposed approach are presented in the sequel together with the collected results. Finally, after a brief presentation of the related works, we draw our conclusions and discuss future work.

## 2. Conventional Serial Acquisition

**Figure 1:**
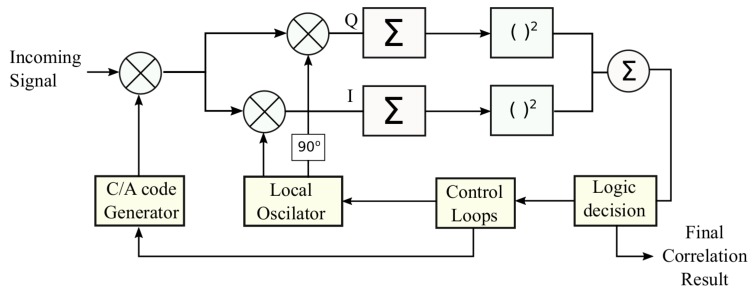
Conventional Acquisition diagram.

The conventional Serial Acquisition (SA) is the simplest acquisition method and is commonly used in hardware GNSS receivers. The basic idea is to test all possible alignments of each satellite’s Pseudo Random Noise (PRN) code and their signal’s carrier frequency between an incoming signal and its locally generated or stored replica. The signal is acquired when there is a match in the code phase and carrier frequency of both signals. This match is detected when the correlation energy between the signals reaches a predetermined threshold (λ), which depends on the level of noise expected. The mathematical expression for this correlation energy is given by

(1) $$\begin{array}{c} R_{C_{f,\phi }}=\sum_{j=0}^{J-1}\left({\left(\sum_{n=jLN}^{(j+1)LN-1}C_{a}^{\phi }\left(n\right)S\left(n\right)\cos\left(2\pi \frac{f}{f_{s}}n\right)\right)}^{2}+{\left(\sum_{n=jLN}^{(j+1)LN-1}C_{a}^{\phi }\left(n\right)S\left(n\right)\sin\left(2\pi \frac{f}{f_{s}}n\right)\right)}^{2}\right),\\ \forall f\in \left\{f_{c}+\delta_{\mathrm{SA}}\left(x-\frac{N_{b}^{\mathrm{SA}}}{2}\right)|x\in I,x\;<N_{b}^{\mathrm{SA}}\right\},\;\forall \phi =\left\{y|y\in I,y\;<1023P\right\}, \end{array}$$

where S(n) is the incoming signal, $f_{c}$ is the central frequency used to transmit the signal, *f* is the probing frequency, $C_{a}^{\phi }\left(n\right)$ is the satellite’s PRN code with phase ϕ, $f_{s}$ is the constant sampling frequency, $N_{b}^{\mathrm{SA}}$ is the number of frequency bins of the SA, $\delta_{\mathrm{SA}}$ is the bandwidth of each of those bins, *N* is the number of samples per code period, $P=\frac{N}{1023}$ is the number of samples per chip, *L* is the number of code periods, and *J* represents the number of signal blocks considered in the calculation of the correlation energy. Each *j*-th block consists of NL signal samples. Figure 1 illustrates the acquisition process.

When the incoming signal and its local replica are aligned in ϕ, the multiplication of these two signals will strip off the PRN code from the incoming signal, resulting in a continuous wave without the code modulation. After the code removal, the signal is multiplied by two carriers, which generates the in-phase (I) and quadrature (Q) components. The I and Q components are then integrated over *L* code periods and the accumulated value in both components are squared and then summed up to each other. This process can be done *J* times to produce averaged correlation values. The last step is to compare the correlation energy value of each code phase and carrier frequency combination with a threshold. If this energy value crosses the λ threshold, the signal is considered acquired. Otherwise, the process is repeated for the next replica with a different combination of phase and frequency.

**Figure 2:**
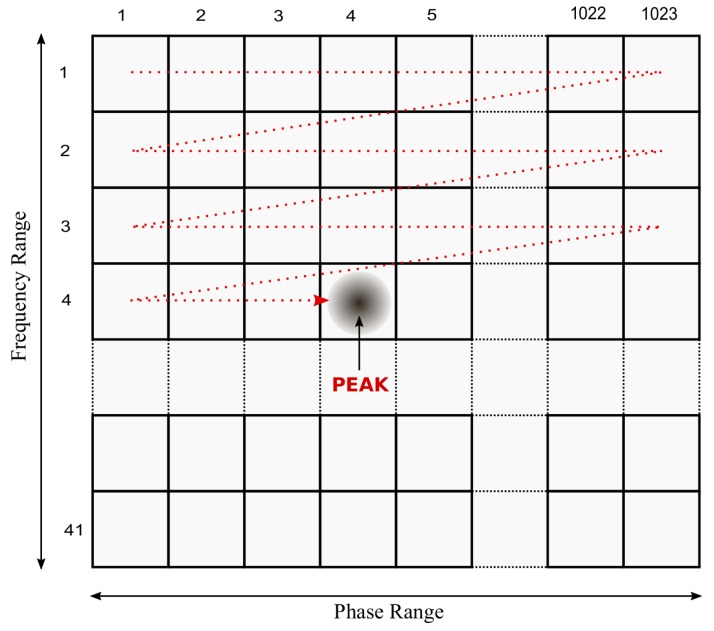
Conventional Acquisition matrix.

Computationally speaking, the I and Q components are represented by two vectors. Each element of these vectors need to be multiplied by the corresponding element of the PRN replica, then accumulated, squared and, finally, the squared values of the I and Q components are added up. Due to the quasi-orthogonality of the PRN codes, if the phases of the incoming signal and its replica are not completely aligned, this final value is very close to zero; otherwise, it peaks [15]. This process is computationally complex due to a large number of different combinations of phase and frequency to be tested for each satellite. Each combination defines a cell for which a new replica needs to be generated and correlated with the incoming signal. The computational complexity of the SA algorithm depends upon the size of the matrix illustrated in Figure 2, the number of samples per chip and the number of integrations.

Since the number of possible code phase shifts in GPS is fixed in 1023, the complexity for those signals can vary according to the frequency offset caused by Doppler effect, i.e., the size of the subset of frequencies in the range around the central frequency, and according to the sampling frequency. When L>1, i.e., when more than 1ms of the signal is used, the acquisition of weak signals improves, but the computational complexity increases. In this paper, we have fixed L=1.

Although slower than methods that perform acquisition in the frequency domain, the SA is quite advantageous in terms of hardware requirements. It requires basically integer multipliers and adders, as Figure 1 shows. This is especially helpful in embedded systems and/or those systems restricted by energy consumption. This is the main rationale for research that aims to improve SA’s overall performance.

## 3. Variance-Triggered Two-Step Acquisition

The technique proposed in this paper, denominated Variance-Triggered Two-Steps (VTTS) acquisition, was first drafted and evaluated in [12,13] with the objective of reducing acquisition time approach without compromising its modest hardware requirements of the conventional serial.

The VTTS acquisition is composed of a Coarse Search (CS) step and a Fine Search (FS) step. In the CS step, the goal is to identify a promising search region in a space with low-frequency resolution by incrementally updating the correlation values of each cell in the search space, sample by sample. The incremental update causes a gradual increase in the correlation values of all cells. By looking for larger variations of these values, it is possible to detect the growing peaks prematurely and then identify a small neighbourhood around it as a promising region, according to the magnitude of these variations. Section 3.1 presents the details about the CS step.

The FS step is used to validate the hypothesis of the signal’s frequency and phase being within the region identified by the CS step. The search in this step is performed using the full resolution. The higher accuracy achieved within this region allows for the validation of the hypothesis at lower computational cost than seeking the same accuracy for the whole search space. True peaks will raise distinguishing themselves from the other cells in the region whereas false peaks will fade down. If the peak value is validated by the FS step, the search procedure ends with an acquired satellite. Otherwise, the CS step restarts from where it was interrupted. Section 3.2 presents the details about the FS step.

The detection of a peak is performed using the variance of the two largest normalized peaks. During the incremental accumulation of the correlation values, a set with the largest peaks values is kept. After each complete iteration in the coarse search space, the highest and the lowest of these peaks are normalized and their variance is calculated. This variance is compared to a threshold of $\lambda_{CS}$ in the CS step. In the FS step, the variance is compared to a different threshold, $\lambda_{FS}$. Both threshold values and the number of peaks kept are design parameters of the receiver. Since the variance values compared to these thresholds are calculated with normalized peak values, this threshold is less sensitive to the noise present in the signal, as indicated by the results in this paper. The details about the detection of the peaks are given in Section 3.3.

### 3.1. The Coarse Search Step

The proposed strategy computes partial correlation values for all cells of the search space using a group of interleaved Subsequent samples in such a way that one sample per chip is used at a time. Although computationally equivalent to when all samples are used, this scheme is considerably different from the conventional acquisition, which calculates the energy of each cell at a time using all samples and visiting each cell only once per phase and frequency pair. The proposed scheme allows for a reduction in the number of samples needed to acquire the signal, as detailed in Section 3.1.1.

Another strategy that reduces computational complexity is applied to the frequency search, reducing the number of frequency bins in the coarse search. Consequently, the bandwidth of each bin is enlarged, reducing accuracy in this step, which is reestablished in the FS step. The rate of reduction in the number of bins is a design parameter of the receiver and should depend mainly on its application domain. The details of this dimensionality reduction strategy are presented in Section 3.1.2.

The correlation values $R_{C_{\hat{f},\hat{\phi }}}$ of the cells with probing estimated frequency $\hat{f}$ and probing estimated phase $\hat{\phi }$ in the coarse search are incrementally computed using 1 millisecond of the signal, i.e., with L=1, as follows.

(2) $$\begin{array}{ccc} R_{C_{\hat{f},\hat{\phi }}}^{d+1} & = & R_{C_{\hat{f},\hat{\phi }}}^{d}+\sum_{\begin{array}{c} n=d\\ \mathrm{steps}\;\mathrm{of}\;P \end{array}}^{N-1}{\left(C_{a}\left(\mathrm{mod}\left(\frac{n}{P}+\hat{\phi },1023\right)\right)S\left(n\right)\cos\left(2\pi \frac{\hat{f}}{f_{s}}n\right)\right)}^{2}+\\ & & {\left(C_{a}\left(\mathrm{mod}\left(\frac{n}{P}+\hat{\phi },1023\right)\right)S\left(n\right)\sin\left(2\pi \frac{\hat{f}}{f_{s}}n\right)\right)}^{2},\\ \forall \hat{f} & \in & \left\{f_{c}+\delta_{\mathrm{CS}}\left(x-\frac{N_{b}^{\mathrm{CS}}-1}{2}\right)|x\in N,x\;<N_{b}^{\mathrm{CS}}\right\},\\ \forall \hat{\phi } & \in & \left\{y|y\in N,y\;<1023\right\},\\ R_{C_{\hat{f},\hat{\phi }}}^{-1} & = & 0, \end{array}$$

where *S* is the vector with *N* samples of the incoming signal, $P=\frac{N}{1023}$ is the number of samples per chip, *d* is the displacement of the interleaved samples in the range [0,P−1], $f_{s}$ is the sampling frequency, $f_{c}$ is the central frequency used to transmit the signal, $N_{b}^{\mathrm{CS}}$ is the number of frequency bins of the CS step, which should be an odd number to include $f_{c}$ in the middle of the range, and $\delta_{\mathrm{CS}}$ is the bandwidth of each of those bins. The vector $C_{a}$ contains the sequence of the 1023 chips for the given satellite. The code $C_{a}$ is shifted by the probing phase $\hat{\phi }$, wrapped around over the 1023th chip, and then multiplied by the incoming signal *S* modulated in phase and in quadrature. The in-phase and quadrature multiplication are then each squared and then accumulated. With the proposed strategy, a coarse correlation peak rises gradually around the searched cell upon each partial increment of Equation (2).

The pseudo code of the CS procedure is listed in Algorithm 1.
**Algorithm 1:** Coarse Search
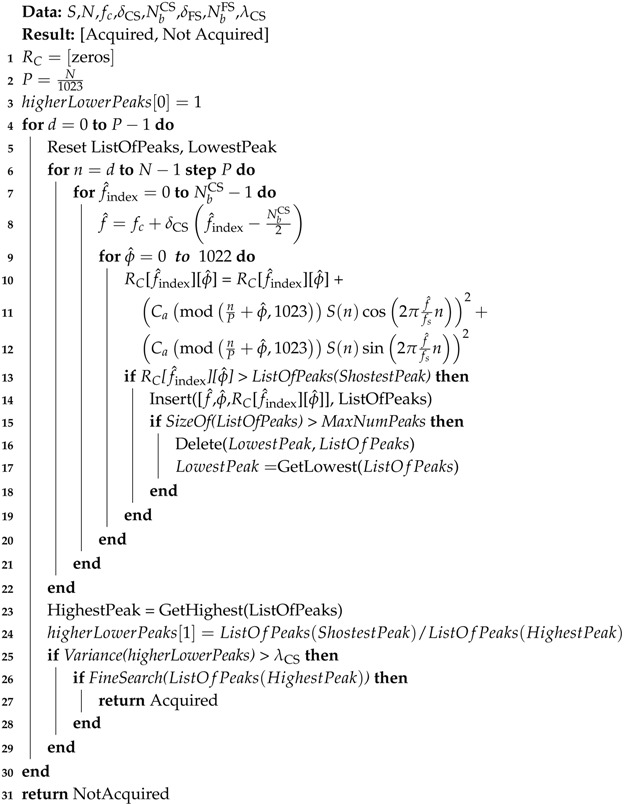


After 1023 interleaved samples are accounted in the second loop, an attempt is made to detect a peak. When a peak is detected, the FS step is triggered. If the satellite is not acquired in the fine search, the next 1023 consecutive interleaved samples are then used to accumulate more samples to the correlation values before the next peak detection. This process is repeated until the satellite is acquired; or until all samples per chip are used, with the given satellite not acquired.

Due to the incremental use of samples, this two-step approach does not have a fixed computational complexity. Each signal requires a different number of operations depending on the quality of the signal. Naturally, low C/N0 signals need more processing.

#### 3.1.1. Reduced Number of Samples

**Figure 3:**
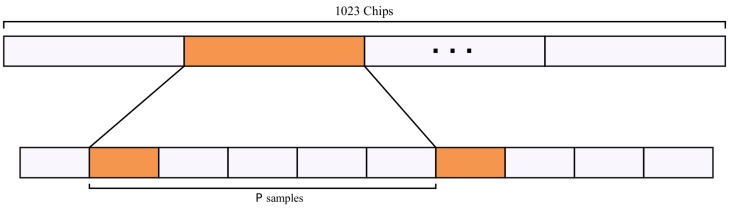
An illustration of chip folding.

In the search for the PRN phase, even a unitary phase misalignment would result in near-zero correlation values due to the auto-correlation properties of the PRN codes. So, it is impractical to reduce the number of phase shifts to be searched for [15]. Nonetheless, it is possible to reduce the number of accounted chip samples and still have useful correlation values. As a consequence of the use of Direct Sequence Spreading Spectrum (DSSS), GNSS signals can be expressed with fewer samples [7]. The signal can be folded and a correlation performed in the folded signal. Figure 3 illustrates this.

**Figure 4:**
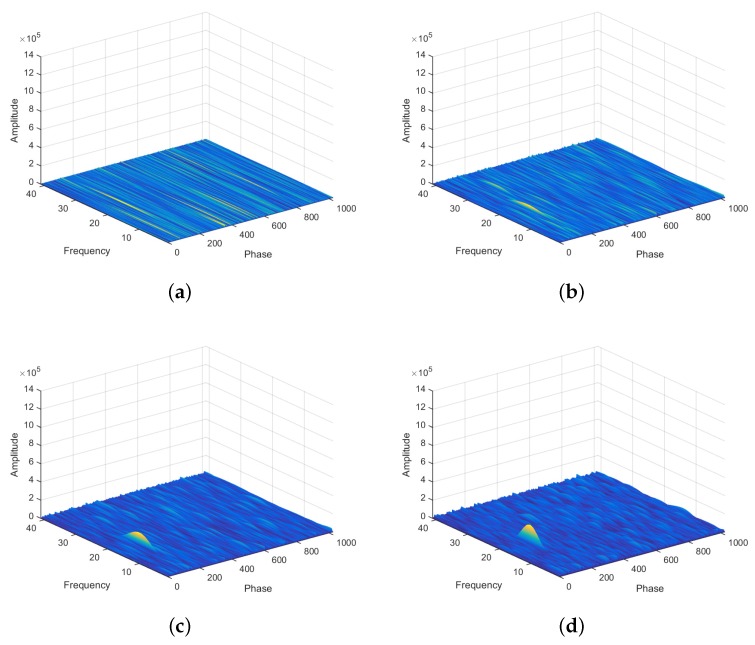
Illustration of the values of a correlation matrix for a satellite search after 4 consecutive rounds of 1023 samples, with the number of samples increasing from (**a**–**d**).

The proposed VTTS approach uses interleaved samples to allow the correlation values to be composed using only one sample at a time from all prospective chips of the signal. Each code chip can be associated with *P* samples but only one is used per chip to accumulate convolution values to the whole search matrix. For that, every other *P* samples are chosen, where *P* is the number of samples per chip. The detection threshold is verified after 1023 interleaved samples. In Figure 4a–c it is possible to observe a peak rising gradually with the increase in the number of interleaved samples.

In the proposed VTTS acquisition, when a peak raises sufficiently to be detected—see Section 3.3, a validation step is initiated to confirm the presence of a satellite signal with characteristics similar to the detected peak—see Section 3.2.

#### 3.1.2. Reduced Number of Frequency Bins

To further reduce the search complexity in the CS step, the number of frequency bins used to correlate the signal across the whole Doppler frequency range is reduced, i.e., $\delta_{CS}>\delta_{FS}$. As a result, the computational complexity of the frequency loop of the CS step decreases with the ratio of $\frac{\delta_{CS}}{\delta_{FS}}$.

**Figure 5:**
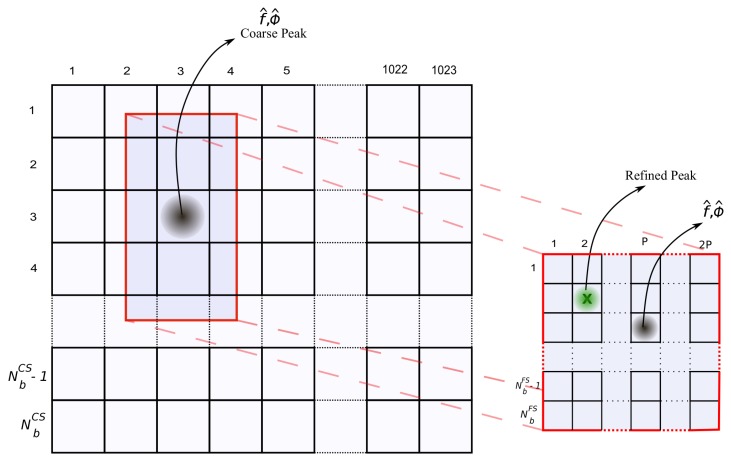
Two-step Acquisition matrix.

Despite the advantages of computational complexity, decreasing the number of bin reduces precision in the estimation of the Doppler frequency shift. Nevertheless, the lost precision is reestablished in the FS step with the use of narrower frequency bins. Figure 5 shows the relationship between the frequency bin width in the CS step and in the FS step. The additional memory complexity added due to this second matrix is very small. In Section 4, we detail the memory usage.

**Figure 6:**
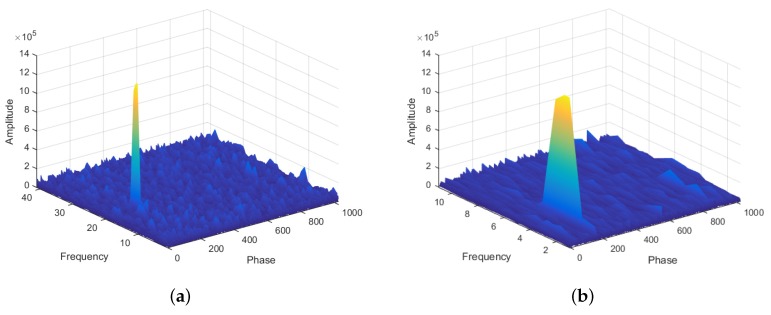
Illustration of the final result of a correlation matrix for a satellite search with (**a**) a fine frequency bin (500 Hz) and (**b**) a coarse frequency bin (2 kHz).

Although GPS theory identifies that the typical bin width should be 667 Hz for the L1 C/A code, in the example of Figure 6, we intentionally used a larger bin width to show that even for those large bins, it is possible to identify a promising frequency region. The case with fine bins has 40 bins of 500 Hz and the case with coarse bins has 10 bins of 2 kHz. Observe that the peak can still be noticed even though with rough precision.

### 3.2. The Fine Search Step

Every time a peak is detected in the CS step, the FS step is triggered. The FS step performs a more precise search for the phase and frequency pair in a confined region of the search space around the pair estimated in the CS step.

Besides the reduced search region, there are other two key differences between the CS step and the FS step. One difference is that while in the CS step the whole Doppler frequency range is completely covered, in the FS step only a small region around the prospecting peak is covered, namely the region between the two frequency bins around the probing frequency. Another key difference is that the search in the phase dimension is reduced to the refinement of the estimated phase using only a few intra-phase displacements, i.e., only 2P phase shifts are searched for.

The starting point of the fine search is the pair of phase $\hat{\phi }$ and frequency $\hat{f}$ estimated in the CS step that triggered the FS step. The search proceeds looking for intra-phase shifts and for a more precise estimation of the frequency shift around $\hat{f}$. The correlation value $R_{C_{f,\phi }}$ of the cells with probing frequency *f* and probing phase ϕ in the fine search using 1 millisecond of signal is defined by

(3) $$\begin{array}{ccc} R_{C_{f,\phi }} & = & \sum_{n=0}^{N-1}{\left(C_{a}\left(\mathrm{mod}\left(\frac{n}{P}+\phi ,1023\right)\right)S\left(n\right)\cos\left(2\pi \frac{f}{f_{s}}n\right)\right)}^{2}+\\ & & {\left(C_{a}\left(\mathrm{mod}\left(\frac{n}{P}+\phi ,1023\right)\right)S\left(n\right)\sin\left(2\pi \frac{f}{f_{s}}n\right)\right)}^{2},\\ \forall f & \in & \left\{\hat{f}+\delta_{\mathrm{FS}}\left(x-\frac{N_{b}^{\mathrm{FS}}-1}{2}\right)|x\in N,\;x<N_{b}^{\mathrm{FS}}\right\},\\ \forall \phi & \in & \left\{y|y\in N,\;\hat{\phi }-P\leq y\leq \hat{\phi }+P,\;y<1023\right\}, \end{array}$$

where $N_{b}^{FS}$ is the number of frequency bins of the FS step, which should be an odd number to include $\hat{f}$ in the middle of the range, and $\delta_{FS}$ is the bandwidth of each of those bins.

Algorithm 2 implements the FS step described in Equation (3).

**Algorithm 2:** Fine Search

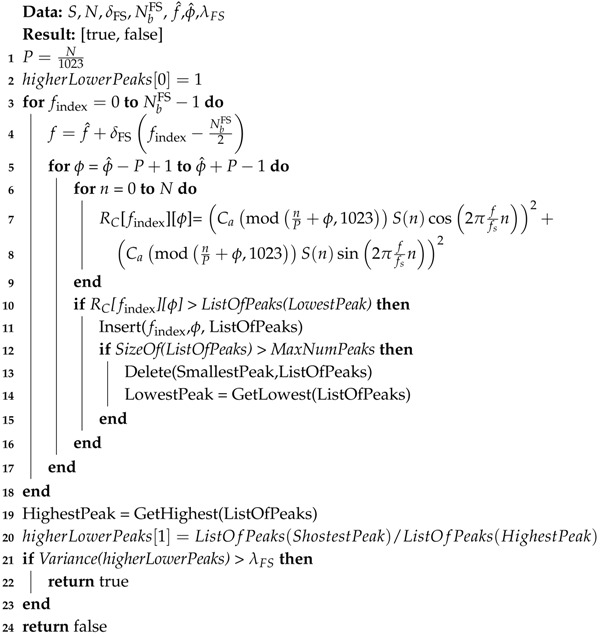



### 3.3. Variance-Triggered Detection Threshold

A key requirement for the proposed approach is the ability to detect correlations peaks that are still forming in the search matrix during the CS step. Conventional fixed-threshold strategies are not appropriate because the detection of each signal behaves differently due to the presence of noise. For signals with higher C/N0, the peak values are higher than for lower C/N0 signals. Using a fixed threshold requires a minimum peak amplitude to avoid false positives, which should be strongly avoided. Consequently, at lower C/N0 signals, this would require integration over longer signal periods to enable peaks to reach the minimum amplitude. Moreover, for the proposed approach, having a fixed threshold does not help when the size of the peaks varies after each detection. Therefore, we developed a method to detect peaks in the CS step based on the variance of the normalized intermediate maximum values of the correlation matrix. For the FS step, the VTTS acquisition uses the same method, but with a different threshold level.

The proposed detection method relies on an ordered list of peaks that is kept updated after the use of every 1023 samples of the signal. In the proposed VTTS acquisition, the number of possible peaks in the CS step is $1023\times N_{b}^{CS}$ and in the FS step it is $2P\times N_{b}^{FS}$. However, only a few peaks are kept in the ordered list. This is necessary to avoid comparing neighbouring secondary peaks that appear under the influence of the main peak, which happens due to the reduced precision and complexity of the CS step. As a chip has *P* samples, to avoid the interference of the neighbouring peaks, we proposed the ordered list to hold only the 3P highest peaks.

#### Defining the Threshold Levels

The GPS acquisition problem is a binary hypothesis test that evaluates the presence or absence of satellites transmitting on the channel. If the null hypothesis 0 ($H_{0}$) is accepted, the analyzed samples are considered to be composed only of noise in relation to a searched satellite. Consequently, as H0 states that the satellite is not present, another satellite should be searched. On the other hand, if the null hypothesis is rejected, hypothesis 1 ($H_{1}$) is accepted and a searched satellite is considered acquired. This binary hypothesis test can be expressed as:

(4) $$\begin{array}{ccc} H_{0} & : & S[n]=W[n] \end{array}$$

(5) $$\begin{array}{ccc} H_{1} & : & S\left[n\right]=W\left[n\right]+C_{a}\left[n\right], \end{array}$$

where S[n] designates the analyzed signal, W[n] represents the noise, and $C_{a}\left[n\right]$ is the signal transmitted by the satellite. Two types of error can occur and cause degradation in the system performance: false-positive and misdetection. A false-positive happens when a satellite is detected in a wrong frequency and phase. This error drives a lost tracking process which, in the end, will not find any data to calculate the position, speed or time. On the other hand, a misdetection occurs when a search satellite is transmitting, but the acquisition process cannot find it. In this case, the acquisition processing time and energy are wasted.

The performance of the detector can be characterized by the error rates, i.e., its probability of false-positive ($P_{F}$) and its probability of misdetection ($P_{MD}$); or equivalently its probability of detection, given by $P_{D}=1-P_{MD}$.

Ideally, $P_{D}=100\%$ and $P_{F}=0\%$, however, usually it is reasonable to accept $P_{D}\geq 90\%$ and a $P_{F}\leq 10\%$ [16], where

(6) $$\begin{array}{ccc} P_{D} & = & P\left(H_{1}|H_{1}\right)=P\left(\wedge >\lambda_{*}|H_{1}\right), \end{array}$$

(7) $$\begin{array}{ccc} P_{F} & = & P\left(H_{1}|H_{0}\right)=P\left(\wedge >\lambda_{*}|H_{0}\right), \end{array}$$

where $\lambda_{*}$ is the detection threshold (CS or FS) and ∧ is the test parameter. In this work, we obtained the threshold by Monte Carlo simulations according to the desired $P_{F}$. In the proposed architecture for GPS acquisition,

(8) $$\begin{array}{ccc} \rho_{1} & = & \frac{\max(ListOfPeaks)}{\max(ListOfPeaks)}=1,\\ \rho_{2} & = & \frac{\min(ListOfPeaks)}{\max(ListOfPeaks)},\\ \wedge & = & \frac{1}{2}\left[{\left(\rho_{1}-\frac{\rho_{1}+\rho_{2}}{2}\right)}^{2}+{\left(\rho_{2}-\frac{\rho_{1}+\rho_{2}}{2}\right)}^{2}\right],\\ \wedge & = & \frac{1}{2}\left[{\left(\frac{1-\rho_{2}}{2}\right)}^{2}+{\left(\frac{\rho_{2}-1}{2}\right)}^{2}\right], \end{array}$$

where ListOfPeaks is the array kept updated with the list of the highest peaks.

The variance threshold for the CS step found empirically was 0.12, while the value found for the FS step was 0.09.

## 4. Memory Usage

This section presents a memory usage analysis for both conventional SA and VTTS acquisition. The memory usage of the SA depends primarily on the number of frequency bins and the number of code phase shifts to be stored in the search matrix. The number of frequency bins is defined by two main receiver design requirements: the maximum Doppler shift, which defines the frequency range ($R_{SA}$) that should be covered by the acquisition; and frequency resolution ($\delta_{SA}$), that is a requirement of the tracking, the next stage of GNSS reception. Given $R_{SA}$ and $\delta_{SA}$, the number of frequency bins in SA can be roughly defined by

(9) $$\begin{array}{c} N_{b}^{\mathrm{SA}}=\frac{R_{\mathrm{SA}}}{\delta_{\mathrm{SA}}}, \end{array}$$

which value should be rounded to the higher odd integer in order to include the central frequency as the middle bin.

The number of code phase shifts depends on another receiver design requirement: the sampling frequency. Together the number of code chips, the sampling frequency will define the number of samples per chip (*P*). Hence, the memory usage for GPS detection using SA is approximated by

(10) $$\begin{array}{c} M_{\mathrm{SA}}=1023PN_{b}^{\mathrm{SA}}. \end{array}$$

For VTTS, the memory usage is composed of the memory used in the two steps of the algorithm,

(11) $$\begin{array}{ccc} M_{\mathrm{VTTS}} & = & M_{\mathrm{CS}}+M_{\mathrm{FS}}, \end{array}$$

(12) $$\begin{array}{ccc} \; & = & 1023N_{b}^{\mathrm{CS}}+2PN_{b}^{\mathrm{FS}}, \end{array}$$

with $M_{\mathrm{CS}}$ and $M_{\mathrm{FS}}$ denoting the memory usage of the CS and FS steps, respectively.

For a fair comparison between the memory usage of SA versus VTTS acquisition, we devise the memory usage of the VTTS method in terms of the same design requirements used for SA. For that, we should guarantee that VTTS delivers the same frequency resolution to the tracking stage, i.e., the resolution of the VTTS’s FS step should be the same as the resolution delivered by SA,

(13) $$\begin{array}{c} \delta_{\mathrm{SA}}=\delta_{\mathrm{FS}}. \end{array}$$

Yet, the same frequency range covered by SA should be covered by VTTS. So, as the range of the SA is defined by the number intervals between bins multiplied by the resolution, we have

(14) $$\begin{array}{c} \left(N_{b}^{CS}-1\right)\delta_{CS}=\left(N_{b}^{SA}-1\right)\delta_{SA}, \end{array}$$

i.e., the range covered by the CS step should be the same covered by the SA. Moreover, we should write Equation (12) in terms of $N_{b}^{\mathrm{SA}}$ and *P*, as in Equation (10), to facilitate the memory usage comparison. However, with more unknown variables than equations, we are obliged to make one assumption to define the number of frequency bins in the FS step. Consider the fact that the resolution of the FS step is always finer than that of the CS step,

(15) $$\begin{array}{c} \delta_{\mathrm{CS}}>\delta_{\mathrm{FS}}, \end{array}$$

and that the frequency range in the FS step is equal to the range between the two bins around the estimated frequency ($\hat{f}$), that is

(16) $$\begin{array}{c} \left(N_{b}^{\mathrm{FS}}-1\right)\delta_{\mathrm{FS}}=2\delta_{\mathrm{CS}}. \end{array}$$

If we assume that Equation (15) must be minimally satisfied, the number of FS bins must be $N_{b}^{\mathrm{FS}}=5$, because a lower odd integer would not satisfy Equation (15), i.e., $N_{b}^{\mathrm{FS}}=3$ means $\delta_{\mathrm{CS}}=\delta_{\mathrm{FS}}$. So, from Equations (13), (14) and (16), and $N_{b}^{\mathrm{FS}}=5$, we have

(17) $$\begin{array}{c} N_{b}^{\mathrm{CS}}=\frac{N_{b}^{\mathrm{SA}}+1}{2}. \end{array}$$

With Equation (17), assuming $N_{b}^{\mathrm{FS}}=5$, we can rewrite Equation (12) in terms of $N_{b}^{\mathrm{SA}}$ and *P*,

(18) $$\begin{array}{c} M_{VTTS}=1023\frac{\left(N_{b}^{\mathrm{SA}}+1\right)}{2}+10P. \end{array}$$

**Figure 7:**
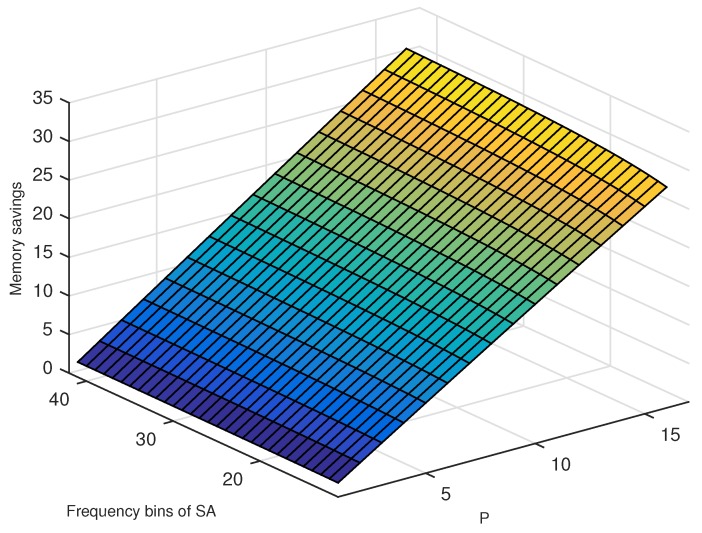
Comparison of memory usage of SA versus VTTS. The depicted values represent the amount of memory savings obtained by VTTS over the SA.

Finally, we can now compare the memory usage of SA and VTTS with the same receiver design requirements. Figure 7 presents the ratio between $M_{\mathrm{SA}}$ (Equation (10)) and $M_{\mathrm{VTTS}}$ (Equation (18)), i.e., the depicted values represent how many times more memory is used by SA regarding VTTS. Values higher than one mean VTTS uses less memory. From the figure, it is clear to see that the memory saving of VTTS regarding SA varies very closely with a linear function of *P*. In other words, if we double the sampling frequency, we would double the memory savings for VTTS.

## 5. Experiments and Results

In this section, we present the results for the proposed VTTS acquisition and four other approaches. One approach refers to the use of the same Two-Step Acquisition (TSA) method of the VTTS acquisition but using the Ratio Detection [14] instead of the proposed variance-triggered detection. This method is called TSA-RD hereafter. The other methods refer to the use of the conventional Serial Acquisition (SA), presented in Section 2, with Fixed Threshold Detection (SA-FTD), with the proposed Variance-Triggered Detection (SA-VTD), and with the Ratio Detection (SA-RD).

The data used in the experiments are GPS L1 band signals with 10 seconds of duration at an intermediate frequency sampled at the rate of 16.368MHz with 2-bit resolution. The signals were obtained using the commercial GNSS simulator Spectracom GSG-5 [17]. All results shown here come from repeating the acquisition 1000 times with a 1-millisecond signal extracted from different random starting points of the signal. We used signals with C/N0 varying between 34 and 50 dB-Hz, which represents a strong signal scenario. The analysis of weak signal scenarios (C/N0 < 34 dB-Hz) should be covered in future work taking into account longer integration times.

The frequency range used in the coarse search was ±10 kHz, which is typically sufficient to cover civil applications [15,18]. The coarse search bin width used was 1kHz and the fine search bin width was 500 Hz. A single fixed variance threshold was used for each of the search steps despite the different C/N0 levels. For the CS step, the threshold was 0.12 while for the FS step it was 0.09.

The following subsections present three different performance aspects of the methods under comparison: the success rate; the rate of false positives; and the computation time necessary to acquire the signal.

### 5.1. Success Rate

**Figure 8:**
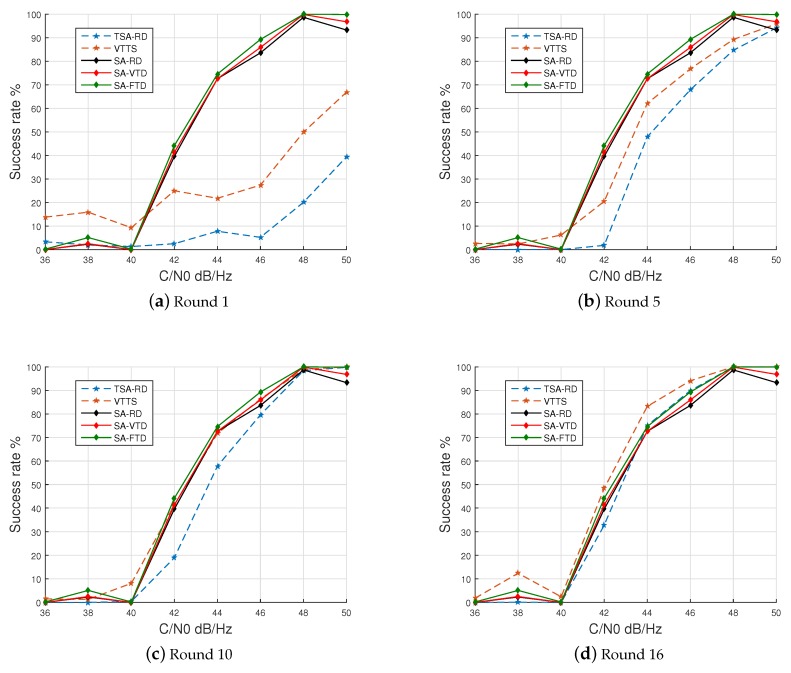
Sucess rate.

The success rates of all algorithms for different C/N0 rates are presented in Figure 8. For VTTS and TSA-RD algorithms, which features the proposed two-step approach and different adaptive threshold detection, the results are presented for 1, 5, 10 and 16 rounds of the CS step. Since the signal being acquired has 16 samples per chip, using 16 rounds of the CS step is the maximum processing that the two-step algorithms can perform although that does not mean all samples were processed. Since the CS step only processes a limited number of samples, some sample will only be processed by the FS step if a promising region is identified in the CS step.

Figure 8 shows that after 16 rounds, the success rate of the VTTS acquisition suppresses those of all other approaches. Moreover, to reach a success rate equivalent to those of the three SA-based approaches, VTTS needs only 10 rounds of the CS step.

### 5.2. False-Positives Rate

The rate of false positives was measured for all algorithms for different levels of C/N0. For the VTTS acquisition, the false-positive rate was measured for 1, 5, 10, and 16 rounds.

**Figure 9:**
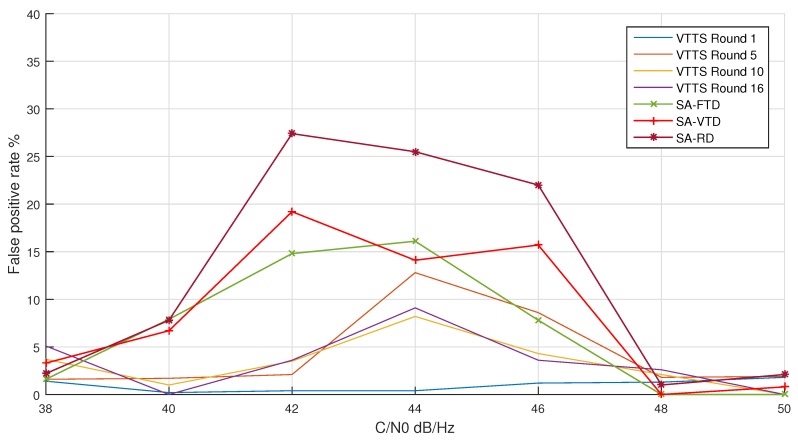
False-positive rate between the three SA versions and the VTTS acquisition for 1, 5, 10 and 16 rounds.

The VTTS technique achieved the lowest false-positive rates, as can be seen in Figure 9. In the worst case, the false-positive rate was about 15% after one round of the CS step. For several rounds larger than 7, this value was always lower than 10%. Overall, the false-positive rates of the VTTS acquisition were always better than the rates of the SA algorithm.

### 5.3. Time to Acquisition

In the previous two Subsections, we have shown that the VTTS acquisition was equivalent or superior to the other four algorithms. In this Subsection, we present other important results of the proposed VTTS method. We show that additional to presenting better success and false-positive rates, it does so with much shorter processing time.

**Figure 10:**
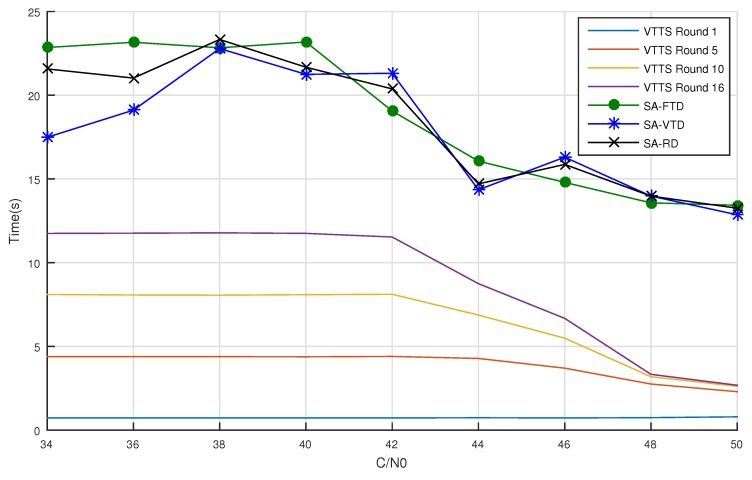
Time to detection for the three SA versions and VTTS acquisition with the maximum number of detection rounds equal to 1, 5, 10 and 16.

We measured the average acquisition time of the three SA versions and the VTTS after 1, 5, 10 and 18 rounds. Figure 10 present those measurements for increasing C/N0 values.

For C/N0 values lower than 42 dB-Hz, the time to acquisition remained constant for the VTTS, which is due to no peak being detected in the CS step. All SA versions had much larger processing times than VTTS because, although the signal is too weak regarding the noise, all samples are processed before skipping the probing satellite.

Starting from a C/N0 of 42 dB-Hz, all algorithms begin to improve average acquisition time. For VTTS, acquisition time reduces at a higher rate for higher quality signals.

The performance gain of VTTS in comparison to SA-FTD in C/N0 rates lower than 44 dB-Hz varies from 49.4% to 96.9%, depending on the number of rounds used. In this case, the detection probability is very small for both algorithms. Spending less time in this situation is much desired to avoid waste of important resources, such as energy in battery powered devices. From C/N0 = 44 dB-Hz on, success rate starts increasing and the performance gain of the VTTS varies between 45.9% and 94.1%. Less time in this situation means faster Time To First Acquisition (TTFA). Table 1 presents average acquisition times depicted in Figure 10.

## 6. Related Works

Time-domain algorithms seek to improve detection requirements like success rate and acquisition time mainly by increasing the length of the observed signal and then folding or sub-sampling it [19,20]. The longer signal length improves accuracy at lower C/N0 ratios because the averaging process reduces noise influence. These strategies, however, iterate over the phase and frequency cells differently than the proposed VTTS strategy. Since the correlation peaks raise incrementally in the VTTS acquisition, the detection of peaks may happen while they are still forming. This is the main difference between the existing time-domain acquisition techniques and the VTTS acquisition.

Increasing integration time is recurrently part of the most effective approaches to increase the success rate. In practice, the existing strategies may always present a trade-off between success rate and acquisition time. We have chosen to not exploit this trade-off in this work, leaving this for a forthcoming investigation. Instead, we have shown that even with only 1 millisecond of the signal, equivalent to a single complete integration over the 1023 chips, the VTTS approach can be quite effective. Longer integration periods would certainly improve VTTS accuracy, as it is the case for the other time domain methods.

This work is considerably different from a preliminary version of the proposed strategy, as presented in [12]. In this work we introduce the mathematical support, an unprecedented memory usage analysis, and a more thorough analysis of success rate, false positive rates, and time to acquisition that involved several levels of C/N0 ratio.

The proposed variance-triggered detection differs from another existing method that does not use a fixed correlation-value threshold to detect the presence of a signal. While the proposed technique uses the variance of normalized peaks, the Ratio Detection (RD) [14] detects signal power based on the ratio of the largest and the second largest correlation value of an incoming signal. In the RD, when the ratio between the peak values exceeds a selected threshold, the signal is considered acquired. In both methods, if more than one peak is selected inside the same chip, the detection algorithm complicates slightly because they have almost the same energy. To solve that, our technique uses the normalized variance of the highest and the lowest peaks from a list of the highest peaks. The RD technique excludes the adjacent samples to the highest peaks.

Other time-domain acquisition methods approach the problem of recovering signals from highly incomplete measurements [7,21]. Due to the necessity of using at least two large matrices, the memory complexity of those methods increase, which can be a limitation for some applications. On the other hand, the proposed VTTS technique uses two small matrices of size $1023\times N_{b}^{\mathrm{CS}}$ and $2P\times N_{b}^{\mathrm{FS}}$. As shown in Section 4, the VTTS has lower memory requirements.

On the phase search domain, a few other techniques also exploit variations in the number and size of frequency bins [11,22]. Differently from this work, in [13], an adaptive strategy for refining the width of the Doppler bins focusing on hardware implementations was investigated, but the VTTS approach proved to work better with the use of large fixed bin sizes at the CS step rather than with an adaptive approach.

Strategies that use two steps like in the proposed VTTS have been proposed in the frequency domain. In [5,6], the authors apply a folding technique on the frequency domain and the coherent integration time is extended to enhance detection performance and, indirectly, reduce mean acquisition time. In the Multi-Peak Double-Dwell [3] approach, a first step selects multiple peaks as candidates; in the second step, the candidate whose signal-to-noise ratio is enhanced by the longer time integration is selected.

In the time domain, following the two-step strategy, a Binarized Convolution Neural Network (BCNN) [23] was used to reduce the search space in a first step, dealing with a long integration time in a reduced search space in a second step [24].

As frequency-domain techniques require supplementary hardware and the BCNN-based approach is quite unconventional and experimental, we consider that comparing these methods to the proposed VTTS acquisition is unsuitable for this work but may be targets for future research.

## 7. Conclusions

This paper presents the Variance-Triggered Two-Step (VTTS) algorithm, a robust method for the efficient acquisition of GNSS signals. It is based on strategies that explore the signal characteristics to reduce acquisition time without increasing the hardware requirements. Additionally, the new technique uses less memory than the conventional approach in the proportion of the number of samples per chip, i.e., the higher the sampling frequency, the larger the memory saving offered by the VTTS acquisition.

Results of extensive experiments with simulated GPS signals with different levels of noise demonstrated that the proposed method presents lower false positive rates than the conventional Serial Acquisition (SA) with slightly better success rate, both at low and high levels of noise. Moreover, the time to acquisition of the VTTS algorithm is about 1/5 of the SA for similar levels of success rates. The VTTS acquisition achieves a comparable success rate already with 10 rounds, as Figure 8c shows. With 16 rounds, the VTTS presents a superior success rate for all C/N0 ratios, except for very high ratios where all methods reach the maximum success rate, as seen in Figure 8d. Moreover, as Figure 10 shows, the VTTS method with 16 and 10 rounds is much faster than all other methods for all levels of C/N0 ratios.

The levels of improvement in success and false-positive rates and in acquisition time were possible due to a variance-triggered detection threshold that allows arbitrary-size correlation peaks to be detected incrementally during a Coarse Search (CS) step. Although the CS step of the VTTS acquisition may generate false-positive overhead working at lower resolutions to reduce computational complexity, the final success rate is not affected or even improves, when compared to the conventional SA, because a Fine Search (FS) step follows the CS step to ensure higher search resolution.

As future work, we pretend to analyze the robustness of the proposed method for longer integration periods, to explore its intrinsic parallelism to further improve acquisition time, to extend the approach to other types of GNSS signals, and to develop a working prototype system with a radio-frequency front-end for use with real signals.

## Figures and Tables

**Table 1:** This table represents the time to acquisition vs C/N0 for the three SA versions and VTTS acquisition with four different rounds (1, 5, 10, 16). The performance of the algorithm was superior in all cases. For round = 1 the VTTS was up to 21 times faster. However, the success rate for this round = 1 is worse than the SA. A better comparison scenario is from round = 5. In this scenario the VTTS was 5.8 times better than the SA in the best C/N0 and 3.7 times better in the worst C/N0 analyzed. When we used round = 16, the gains were 1.85 and 5.05 in the worst and best C/N0 respectively.

	C/N0 (dB-Hz)								
	34	36	38	40	42	44	46	48	50
	Acquisition Time (s)								
SA-FTD	22.80	23.16	22.83	23.18	19.05	16.07	14.79	13.57	13.43
SA-VTD	17.49	19.10	22.78	21.31	14.35	14.36	16.30	13.98	12.86
SA-RD	21.57	21.02	23.33	21.67	20.37	14.70	15.87	13.97	13.25
VTTS 1 round	0.73	0.73	0.73	0.72	0.72	0.74	0.72	0.74	0.79
VTTS 5 rounds	4.39	4.38	4.38	4.38	4.40	4.28	3.70	2.70	2.20
VTTS 10 rounds	8.10	8.06	8.05	8.08	8.11	6.87	5.49	3.19	2.68
VTTS 16 rounds	11.70	11.76	11.78	11.74	11.53	8.70	6.60	3.30	2.66
